# The Relation of Rapid Changes in Obesity Measures to Lipid Profile - Insights from a Nationwide Metabolic Health Survey in 444 Polish Cities

**DOI:** 10.1371/journal.pone.0086837

**Published:** 2014-01-31

**Authors:** Bernhard M. Kaess, Jacek Jóźwiak, Christopher P. Nelson, Witold Lukas, Mirosław Mastej, Adam Windak, Tomasz Tomasik, Władysław Grzeszczak, Andrzej Tykarski, Jerzy Gąsowski, Izabella Ślęzak-Prochazka, Andrzej Ślęzak, Fadi J. Charchar, Naveed Sattar, John R. Thompson, Nilesh J. Samani, Maciej Tomaszewski

**Affiliations:** 1 Department of Cardiovascular Sciences, University of Leicester, Leicester, United Kingdom; 2 Deutsches Herzzentrum, Technische Universität, Munich, Germany; 3 Department for Internal Medicine II, University Hospital Regensburg, Regensburg, Germany; 4 Department of Public Health, Częstochowa University of Technology, Częstochowa, Poland; 5 Silesian Analytical Laboratories, Katowice, Poland; 6 The NIHR Leicester Biomedical Research Unit in Cardiovascular Disease, Leicester, United Kingdom; 7 Department of Family Medicine, Medical University of Silesia, Zabrze, Poland; 8 Department of Family Medicine, Jagiellonian University Medical College, Kraków, Poland; 9 Department of Internal Diseases, Diabetology and Nephrology, Medical University of Silesia, Zabrze, Poland; 10 Department of Hypertension, Vascular Diseases, and Internal Diseases, University of Medical Sciences, Poznań, Poland; 11 Department of Internal Medicine and Gerontology, Jagiellonian University Medical College, Kraków, Poland; 12 School of Health Sciences, University of Ballarat, Ballarat, Australia; 13 Institute of Cardiovascular and Medical Sciences, University of Glasgow, Glasgow, United Kingdom; 14 Department of Health Sciences, University of Leicester, Leicester, United Kingdom; University of South Florida College of Medicine, United States of America

## Abstract

**Objective:**

The impact of fast changes in obesity indices on other measures of metabolic health is poorly defined in the general population. Using the Polish accession to the European Union as a model of political and social transformation we examined how an expected rapid increase in body mass index (BMI) and waist circumference relates to changes in lipid profile, both at the population and personal level.

**Methods:**

Through primary care centres in 444 Polish cities, two cross-sectional nationwide population-based surveys (LIPIDOGRAM 2004 and LIPIDOGRAM 2006) examined 15,404 and 15,453 adult individuals in 2004 and 2006, respectively. A separate prospective sample of 1,840 individuals recruited in 2004 had a follow-up in 2006 (LIPIDOGRAM PLUS).

**Results:**

Two years after Polish accession to European Union, mean population BMI and waist circumference increased by 0.6% and 0.9%, respectively. This tracked with a 7.6% drop in HDL-cholesterol and a 2.1% increase in triglycerides (all p<0.001) nationwide. The direction and magnitude of the population changes were replicated at the personal level in LIPIDOGRAM PLUS (0.7%, 0.3%, 8.6% and 1.8%, respectively). However, increases in BMI and waist circumference were both only weakly associated with HDL-cholesterol and triglycerides changes prospectively. The relation of BMI to the magnitude of change in both lipid fractions was comparable to that of waist circumference.

**Conclusions:**

Moderate changes in obesity measures tracked with a significant deterioration in measures of pro-atherogenic dyslipidaemia at both personal and population level. These associations were predominantly driven by factors not measureable directly through either BMI or waist circumference.

## Introduction

Clinical measures of obesity such as body mass index (BMI), waist circumference and waist/height ratio are strong risk factors of cardiovascular and metabolic diseases [1]–[4]. The central role of obesity in cardiovascular disorders is undisputed [5]. It is also widely acknowledged that the effect of obesity on cardiovascular disease is at least partly mediated by its traditional risk factors including lipids [6], [7]. However, the exact mechanisms underlying relationships between BMI/waist indices and circulating levels of lipids remain elusive. In fact, there is a surprising paucity of epidemiological studies that have investigated the impact of changes in abdominal and general obesity measures on lipids. In particular, it is not clear to what extent increasing obesity at the population level is associated with a deteriorating lipid profile. It is also unknown whether increasing visceral obesity (i.e. best approximated simply by waist circumference) at the population levels may have a stronger relationship with deteriorating lipid profile than changes in general obesity (assessed by BMI).

To examine the extent of association between changes in measures of obesity and lipids we took advantage of data collected in an exceptional “experiment of history” – the accession of Poland to the European Union in 2004. We hypothesised that the economic and social consequences of this major political development in Europe would lead to rapid changes in measures of obesity and that these changes may have a significant impact on circulating levels of lipids in the Polish population. We chose to quantify and relate the short-term (2-year) changes in both measures of obesity to lipids initially at the population (cross-sectional analysis) and then seek independent replication of the findings at the personal level (prospective level).

## Materials and Methods

### Study Populations

LIPIDOGRAM was a nationwide survey of cardiovascular risk factors carried out through primary care outpatient centres in Poland right after Polish accession to the EU (2004) and two years later (2006). In 2004, a total of 700 doctors in 16 major administrative regions in Poland were invited to recruit up to 30 consecutive adults attending their primary care practices into this study. Between October and December 2004 a total of 675 primary care specialists actively enrolled 17,522 individuals in 444 cities. Of these 15,404 were recruited into the cross-sectional arm (no follow-up; LIPIDOGRAM2004) and 2,118 into a separate prospective observation with a planned follow-up in 2006 (LIPIDOGRAM PLUS).

Between October and December 2006, 556 primary care practitioners (82.4% of those recruiting in 2004) from 402 Polish cities recruited a total of 15,465 individuals into another cross-sectional analysis (LIPIODGRAM2006). In addition, individuals recruited into LIPIDOGRAM PLUS in 2004 had their follow-up in 2006. None of the participants recruited into the cross-sectional analysis in 2004 were included in the population survey in 2006. There was also no overlap in individuals recruited into the cross-sectional and the prospective studies.

The number of recruitment centres across 16 major administrative regions of Poland as well as the number of individuals recruited in each region and included in analysis was a direct function of demographic size of each region (**Table S1**). These proportions were retained in 2004 and 2006 in the cross-sectional surveys as well as in the prospective analysis (**Tables S1–2** and **Figures S1–3**). All recruiting medical practitioners underwent a formal training in the study protocol, procedures and collection of the standardised questionnaires and blood samples.

Eligible for recruitment were individuals aged at least 30 years who attended an appointment in the primary care during the recruitment period. The exclusion criteria were dementia/mental disease resulting in inability to give an informed consent, and incomplete clinical or biochemical information. Each individual was phenotyped according to the protocol introduced in LIPIDOGRAM2004 and described in detail before [2]. In brief, phenotyping included collection of basic demographic and clinical data by standardised coded questionnaires, anthropometric measurements (weight, height, waist circumference) and fasting blood sample for further biochemical analyses. Weight and height measurements were carried out without heavy clothing and shoes. Waist circumference was measured over the unclothed abdomen at a level of the midpoint between the lower margin of the ribs and the anterior superior iliac crest spine [2]. Smoking was defined as current smoking of at least 1cigarette/day. All patients were of white Polish ethnicity.

All participants provided written informed consent. The study was approved by the Bioethical Committee of the Polish Chamber of Physicians (No 51/2004/U) and conforms to the principles outlined in the Declaration of Helsinki.

### Biochemical Analyses

Blood samples were taken under fasting (for at least 12 hours) conditions. Blood samples were centrifuged locally and separated serum samples were transferred by one contracted courier company to the central facility where all biochemical analyses were conducted within 12 hours after blood collection. All biochemical measurements (serum) were conducted in the same International Organization for Standardization (ISO) certified laboratory and under the same experimental conditions on an automatic bio-analyzer (ARCHITECT-c8000, ABBOTT Laboratories, USA). Serum concentrations of total cholesterol were measured using a photometric method with cholesterol oxidase (DiaSys – Diagnostic Systems, Germany). HDL-cholesterol (HDL-C) and triglycerides were analyzed by immuno-separation-based homogenous assay and colorimetric enzymatic test with glycerol-3-phosphateoxidase, respectively (both DiaSys – Diagnostic Systems, Germany). The intra-assay and inter-assay coefficients of variation for total cholesterol, HDL-C and triglycerides were low (0.61, 1.22 and 0.41, 0.83 and 1.23, 1.60, respectively). LDL-cholesterol (LDL-C) was calculated using the Friedewald formula in those whose triglycerides were measured <4.5 mmol/L.

Internal tests of accuracy and precision were conducted by using CDC-certified lyophilised human serum control samples (including those with high, normal and low values for each directly measured lipid fraction) together with participants’ samples in each run. Total error (TE – a measure that combines analytical imprecision and systematic bias) for each of these lipid fractions was calculated using the formula: TE = Bias(%)+2CV and never exceeded the total allowable error calculated at 9% for TC, 14% for TG and 16% for HDL-C. The national external audit of quality control (National Research Centre for Quality in Laboratory Diagnostics, Lodz, Poland) and the international accreditation of the external control (Labquality, Helsinki, Finland and INSTAND e.V., Düsseldorf, Germany) were conducted both in 2004 and 2006 and confirmed good quality standards for lipids measurements.

### Statistical Analysis

Changes in quantitative metabolic variables were analysed using linear models fitted by standard regression to the cross-sectional data and by Generalized Estimating Equations to the prospective data. Triglycerides were log-transformed prior to regression analysis and back-transformed into the original measurement scale (data are presented as geometric means and their standard errors). All models included a term for the survey year (coded as 2004 = 0, 2006 = 1). Fully adjusted regression models included terms for age, age^2^, sex, height, region of recruitment, current smoking and education (coded as primary, secondary or higher).

Sex differences in changes in measures obesity and lipids were examined by comparison of corresponding regression coefficients using Wald (z-statistic) tests.

In the prospective LIPIDOGRAM PLUS study, adjusted and unadjusted tests for linear trend were used to examine association between changes in lipids (as a continuous measure) across categorised change in BMI/waist circumference.

## Results

The general and sex-stratified clinical characteristics of the study groups are summarised in **Table 1** and **Tables S3–4**. A total of 14,849 and 15,453 individuals recruited into the cross-sectional survey in 2004 and 2006 were included in this analysis after excluding those with missing or inconsistent information. A total of 1,840 individuals with full demographic and biochemical data recruited in 2004 into the prospective LIPIDOGRAM PLUS were surveyed again in 2006 (follow-up rate –86.9%).

**Table 1:** General clinical characteristics of LIPIDOGRAM2004, LIPIDOGRAM2006 and LIPIDOGRAM PLUS Studies.

Characteristic	Cross-sectional			Prospective		
	2004	2006	P-value	2004	2006	P-value
**N**	14849	15453	–	1840	1840	–
**Men (%)**	6004 (40.4)	5806 (37.6)	<0.001	712 (38.7)	712 (38.7)	–
**Age (years)**	55.4 (10.7)	55.5 (11.1)	0.46	53.3 (9.7)	55.3 (9.7)	–
**Height (cm)**	166.4 (8.6)	166.1 (8.4)	<0.001	166.7 (8.5)	166.7 (8.5)	–
**Weight (kg)**	78.3 (15.1)	78.5 (14.9)	0.14	78.0 (14.9)	78.9 (15.1)	<0.001
**BMI (kg/m^2^)**	28.2 (4.8)	28.4 (4.6)	<0.001	28.0 (4.6)	28.3 (4.6)	<0.001
**Waist (cm)**	92.4 (13.1)	93.1 (13.0)	<0.001	91.3 (12.5)	92.1 (12.5)	<0.001
**HDL-C (mmol/L)**	1.65 (0.39)	1.53 (0.38)	<0.001	1.68 (0.41)	1.54 (0.38)	<0.001
**TG (mmol/L)**	1.49 (0.69)	1.53 (0.64)	<0.001	1.47 (0.69)	1.52 (0.65)	<0.001
**TC (mmol/L)**	5.76 (1.12)	5.66 (1.13)	<0.001	5.82 (1.15)	5.67 (1.13)	<0.001
**LDL-C (mmol/L)**	3.36 (0.95)	3.37 (0.99)	0.47	3.40 (0.98)	3.38 (0.99)	0.42
**Smokers (%)**	3126 (21.1)	2879 (18.6)	<0.001	361 (19.6)	317 (17.2)	<0.001
**Treatment (%)**	4555 (30.7)	4898 (31.7)	0.06	512 (27.8)	706 (38.4)	<0.001

Data are means and standard deviations, geometric means and standard deviations (triglycerides) or counts and percentages; BMI – body mass index; HDL-C – high-density lipoprotein cholesterol; TG – triglycerides; TC – total cholesterol; LDL-C – low-density lipoprotein cholesterol; treatment – lipid-lowering medication.

In the cross-sectional study adjusted BMI and waist circumference increased between 2004 and 2006 by approximately 0.6% [0.18 (0.05) kg/m^2^, P<0.001] and 0.9% [0.86 (0.14) cm, P<0.001], respectively (**Table 2**). These changes in the population were mirrored at the individual level; BMI increased by 0.7% [0.2 (0.05) kg/m^2^, P<0.001] and waist circumference by 0.3% [0.28 (0.16) cm, P = 0.09] in LIPIDOGRAM PLUS (**Table 2**). The direction of change in both indices of obesity between 2004 and 2006 was similar in both sexes, although the increasing trend was generally more prominent in men (**Table S5**).

**Table 2:** Changes in obesity measures and lipids between 2004 and 2006 in the cross-sectional and prospective LIPIDOGRAM Studies.

Measure	Model	Cross-sectional		Prospective	
		β (SE)	P-value	β (SE)	P-value
**BMI (kg/m^2^)**	Basic	0.20 (0.05)	<0.001	0.33 (0.04)	<0.001
	Full	0.18 (0.05)	<0.001	0.20 (0.05)	<0.001
**Waist (cm)**	Basic	0.72 (0.15)	<0.001	0.89 (0.15)	<0.001
	Full	0.86 (0.14)	<0.001	0.28 (0.16)	0.09
**HDL-C** **(mmol/L)**	Basic	−0.119 (0.004)	<0.001	−0.147 (0.006)	<0.001
	Full	−0.125 (0.004)	<0.001	−0.145 (0.006)	<0.001
**TG (mmol/L)**	Basic	0.027 (0.005)	<0.001	0.034 (0.009)	<0.001
	Full	0.031 (0.005)	<0.001	0.026 (0.009)	<0.001
**TC (mmol/L)**	Basic	−0.104 (0.013)	<0.001	−0.151 (0.025)	<0.001
	Full	−0.100 (0.013)	<0.001	−0.162 (0.025)	<0.001
**LDL-C** **(mmol/L)**	Basic	0.008 (0.011)	0.46	−0.016 (0.022)	0.05
	Full	0.015 (0.011)	0.19	−0.026 (0.022)	0.24

Data are changes between 2004 and 2006 are expressed as β-coefficients with standard errors (SE); BMI – body mass index; HDL-C – high-density lipoprotein cholesterol; TG – triglycerides; TC – total cholesterol; LDL-C – low-density lipoprotein cholesterol; Basic – unadjusted model; Full – model adjusted for age, age^2^, sex, region of recruitment, height, education and smoking.

Of four lipid fractions the most significant change over the two-year observation period was observed in circulating concentrations of HDL-C. After adjustment, HDL-C dropped by 7.6% [0.125 (0.004) mmol/L, P<0.001] nationwide. The increase in circulating concentrations of triglycerides was calculated at 2.1% (P<0.001) (**Table 2**). Serum levels of LDL-C levels remained essentially constant whilst total cholesterol dropped by 1.7% (P<0.001) in the surveyed population (**Table 2**). The direction and magnitude of changes in lipids in the prospective LIPIDOGRAM PLUS were very similar; a 8.6% drop in HDL-C (P<0.001) was accompanied by a 1.8% increase in triglycerides (P<0.001), a 2.8% decrease in total cholesterol (P<0.001) and no changes in LDL-C (**Table 2**). These trends were also apparent in sex-specific analysis with very little difference in the magnitude of the changes in lipids between men and women over the two-year observation period (**Table S5**).

We then conducted a sensitivity analysis by selecting 21,983 subjects who were not on pharmacological lipid-lowering treatment (n = 10,294, n = 10,555 and n = 1,134 individuals in LIPIDOGRAM2004, LIPIDOGRAM2006 and LIPIDOGRAM PLUS, respectively). As expected, after exclusion of patients on therapy, LDL-C showed a small increase between 2004 and 2006 in both the cross-sectional and prospective analysis (**Table S6**). The trends in other lipids in this sensitivity analysis were consistent with those in the entire cross-sectional and prospective samples.

**Figure 1:**
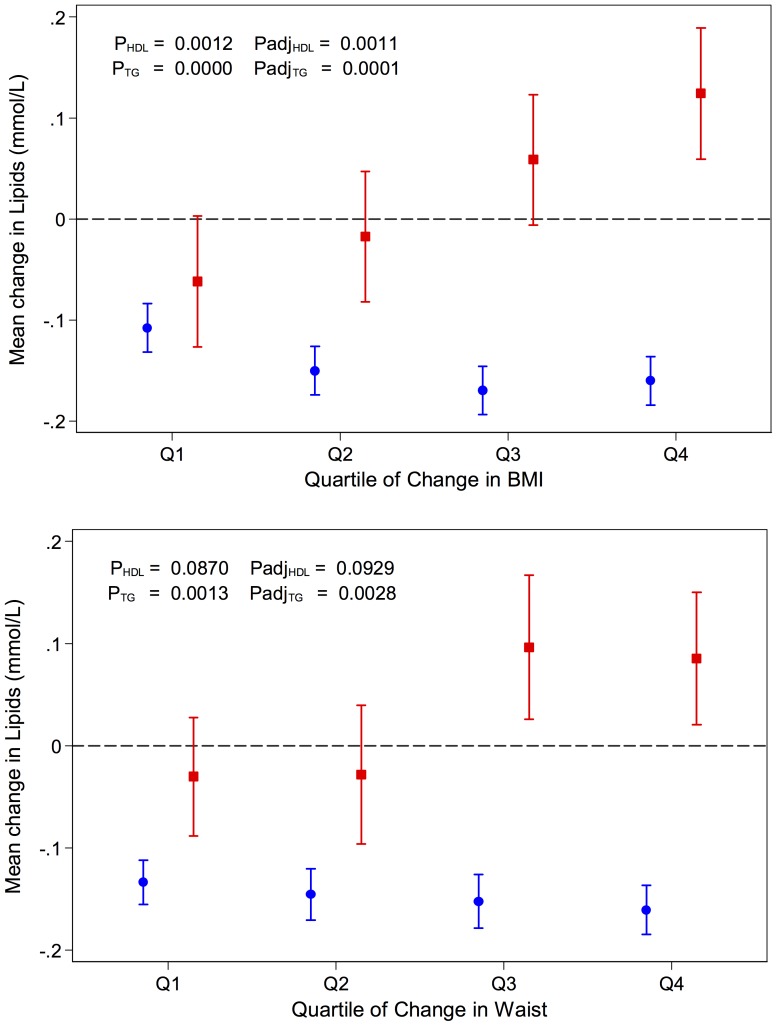
Relation of changes in obesity measures to changes in lipids in the LIPIDOGRAM PLUS Study. Mean changes in HDL-C (blue squares) and triglycerides (red circles) across quartiles of changes in BMI (top panel) and waist circumference (bottom panel) between 2004 and 2006. Data are means and standard errors, the bottom quartile represents subjects from the lowest 25% of distribution in BMI or waist circumference increase between 2004 and 2006. P-values for trend across quartiles; Padj - adjusted (for age, sex, region of recruitment, height, education and smoking) level of statistical significance from test for trend.

Using the data from the LIPIDOGRAM PLUS study we next assessed whether changes in obesity measures were directly related to the observed change in HDL-C, triglycerides, and total cholesterol over the two-year observation period. There was no direct association between changes in either BMI or waist circumference and total cholesterol levels (**Figure S4** and **Table S7**). The linear trends in changes of HDL-C and triglycerides across the quartiles of BMI/waist circumference change distribution (**Figure 1** and **Table S7**) were modest. Changes in waist did not show a greater magnitude of association with HDL-C and triglycerides than BMI. In fact, the statistical significance of the association with changes in lipids was weaker for waist than BMI (**Figure 1** and **Table S7**).

## Discussion

We investigated changes in two indices of obesity and main lipid fractions in a large sample of Polish individuals recruited after a major political and economic transformation in Europe – the accession of Poland to the EU in 2004. Economic growth is one the most important environmental drivers of obesity [8], [9]. Indeed, socioeconomic transformations of the 1990’s exposed the Eastern European populations to food market globalization that promotes processed, high in calories food. Changes in eating cultures and increasing rates of physical inactivity in the absence of appropriate health campaigns may have made this region particularly vulnerable to obesity. We hypothesized that the fast economic transition related to the European integration would provide an excellent epidemiological model to examine the relations of an increasing obesity burden with lipids in the Polish population.

We report several important findings. Firstly, our data demonstrate the expected increase in major anthropometric measures of obesity, tracking with a marked deterioration in HDL-C and triglycerides 2 years after the Polish accession to the EU. Secondly, the relative changes in these lipid fractions were actually greater than those of BMI or waist circumference, and only weakly associated with the increase in indices of obesity. Thirdly, our survey demonstrates that waist circumference and BMI are largely comparable as weak metabolic proxies of changes in lipids in both men and women. Finally, we show for the first time that the changes in obesity measures and lipids observed in the nationwide population are largely reproducible at the personal level.

Very few studies reported how changes in BMI and other measures of obesity translate into changes in levels of lipids in individual patients and populations. An increase in measures of obesity over a period of six–ten years has been associated with a deterioration of all major lipid fractions [10], components of metabolic syndrome [11] and incident hypercholesterolemia [12] in different studies. In contrast, a ten-year observation in the Framingham Heart Study revealed that an overall increase in BMI in the population was accompanied by an unexpected increase in HDL-C and a drop in triglycerides [13]. In our study, a short-term increase in BMI/waist circumference was paralleled by a significant deterioration in triglycerides and most apparently – HDL-C. However, neither the increase in BMI nor the increase in waist circumference could statistically explain the deterioration in HDL-C. This means that the observed drop in HDL-C is to a large extent independent of the parallel increase in BMI or waist circumference. Prevalence of smoking, another well-known correlate of low HDL-C [14] actually decreased between 2004 and 2006; thus it is unlikely to explain our findings. Diet, alcohol consumption and physical activity on their own may also impact on HDL-C and other lipid fractions, independent on their contribution to weight balance [15]–[17]. Insulin resistance and low-grade inflammation that cluster with excessive weight have also been shown to affect HDL-C and/or triglycerides, independent of adiposity [18], [19]. Unfortunately, information on either of these phenotypes was not available in the LIPIDOGRAM project. A significant drop in HDL-C largely unexplained by measures of obesity was reported before in longer observations of general population [20], [21]. These studies relied on BMI as the only measure of obesity. Our data show that these associations are apparent even short-term and that using waist circumference does not offer a better explanation for the change in HDL-C than BMI and that other factors, not captured directly through either of these anthropometric indices, may be responsible here.

Parallel to the significant changes in HDL-C, LDL-C was essentially stable in both the cross-sectional and prospective study. In the absence of a major change in LDL-C levels the (somewhat paradoxical) decrease in total cholesterol tracking with increasing measures of obesity is most likely related to decreasing HDL-C levels. Our data indicate that the observed drop in total cholesterol is not related to the use of statins. Indeed, after exclusion of participants on statin-based treatment, there was an apparent decreasing trend in total cholesterol despite slightly increasing LDL-C.

To the best our knowledge this is the first investigation that has examined changes in metabolic health at both individual and population level simultaneously using identical protocols. The availability of prospective personal-level observation in addition to the two cross-sectional surveys provided also an independent replication. A consistency in the direction and magnitude of metabolic changes in both surveys also suggests that there is a good correlation between personal and nationwide trends in metabolic health. This consistency is also reassuring given that changes in cardio-metabolic risk factors from repeated observational studies are often criticised for providing overestimates due to unmeasured confounding. To this end our data point to common environmental roots of pro-atherogenic dyslipidaemia in individual patients and populations.

It would be fair to acknowledge that our data provide only a snapshot of the changes driven by the long-term process of political, economic and social transformation in Poland initiated by the Polish Solidarity movement in 1980s and transition to market economy in the early 1990s. We also recognise that LIPIDOGRAM surveys were recruited amongst individuals older than 30 years and thus may not be fully representative of the general population structure. The recruitment through primary care may also lead to over-representation of individuals with ill health, although a positive or negative bias towards any particular disease condition is unlikely given the wide inclusion criteria. Furthermore, the LIPIDOGRAM protocols did not include questionnaires on eating habits or physical activity. Finally, our study is observational, and definite causal inferences cannot be made.

However, our analysis has several strengths. Firstly, the recruitment strategy, phenotyping, data collection and processing of all LIPIDOGRAM data were carried out using essentially identical protocols. To minimise heterogeneity between 2004 and 2006 surveys no new recruitment sites were included in LIPIDOGRAM2006 and a majority of recruiting investigators from LIPIDOGRAM2004 participated again in 2006. Secondly, all surveys were recruited at the same time of the year, minimising differences related to seasonal variation i.e. in lipids. Thirdly, all recruiting doctors were trained prior to recruitment to maximise the standardisation of data collection. Furthermore, to ensure appropriate representation of individuals from all parts of the country the number and regional distribution of recruitment sites in LIPIDOGRAM studies was carefully matched to the demographic structure of individual geographic regions of Poland. A unique advantage of our study is that it used a nationwide rather than community samples. Regional variation in obesity measures across one country is a well-recognised source of potential confounding and the recruitment restricted to one community may not necessarily reflect the nationwide trends in obesity changes [7]. Finally, to eliminate inter-laboratory variation in biochemical measurements all blood analyses were conducted in one central laboratory that conforms to all national and international quality control standards. Therefore, we have no reason to suspect that a systematic bias in methodology may have influenced the findings of our study.

## Conclusions

In summary, using data collected in Poland at the time and shortly after its accession to EU we demonstrate a deterioration of metabolic health at both personal and population level. The rapid increase in clinical measures of obesity tracked with a parallel deterioration in measures of pro-atherogenic dyslipidaemia, namely a drop in HDL-C and an increase in triglycerides. However, the magnitude of change in lipids was far greater than that of BMI or waist circumference and either measure of obesity were only weakly associated with changes in lipids. Thus, the observed deterioration in lipid profile was predominantly driven by other factors not measureable through BMI or waist circumference.

## Supporting Information

Figure S1 **Correlation between numbers of individuals recruited in 2004 and 2006 into the cross-sectional LIPIDOGRAM Studies.**
(TIFF)

Figure S2 **Correlation between numbers of individuals recruited in 2004 into the cross-sectional and prospective LIPIDOGRAM Studies.**
(TIFF)

Figure S3 **Correlation between numbers of individuals recruited in 2006 into cross-sectional and prospective LIPIDOGRAM Studies.**
(TIFF)

Figure S4 **Mean changes in total cholesterol across 4 quartiles of changes in BMI (top panel) and waist circumference (bottom panel) between 2004 and 2006 in LIPIDOGRAM PLUS Study.** Data are means and standard errors, the lowest quartile (Q1) - bottom 25^th^ percentile of distribution in BMI or waist circumference increase between 2004 and 2006; P-values – level of statistical significance from test for linear trend; Padj - level of statistical significance (test for linear trend) after adjustment for age, sex, region of recruitment, height, education and smoking, the dotted horizontal line – no change.
(TIFF)

Table S1 **Number of recruiting cities, physicians, recruited and included individuals in cross-sectional LIPIDOGRAM2004 and LIPIDOGRAM2006.** Population data obtained from the Polish National Main Statistical Office (Glowny Urząd Statystyczny; GUS) –2003.
(DOCX)

Table S2 **Individuals recruited into the prospective LIPIDOGRAM PLUS Study.**
(DOCX)

Table S3 **Clinical characteristics of cross-sectional LIPIDOGRAM2004 and LIPIDOGRAM2006 Studies – sex-stratified analysis.** Data are means and standard deviations, geometric means and standard deviations (triglycerides) or counts and percentages; BMI – body mass index; HDL-C – high-density lipoprotein cholesterol; TG – triglycerides; TC – total cholesterol; LDL-C – low-density lipoprotein cholesterol; treatment – lipid-lowering medication; P-value – level of statistical significance for comparison of men versus women.
(DOCX)

Table S4 **Clinical characteristics of LIPIDOGRAM PLUS Study – sex-stratified analysis.** Data are means and standard deviations, geometric means and standard deviations (triglycerides) or counts and percentages; BMI – body mass index; HDL-C – high-density lipoprotein cholesterol; TG – triglycerides; TC – total cholesterol; LDL-C – low-density lipoprotein cholesterol; treatment – lipid-lowering medication; P-value – level of statistical significance for comparison men versus women.
(DOCX)

Table S5 **Changes in metabolic parameters between 2004 and 2006 in the LIPIDOGRAM studies – sex-stratified analysis.** Changes between 2004 and 2006 are expressed as β-coefficients (β) with respective standard errors (SE) from linear regression or generalized estimation equations-based models; BMI – body mass index; HDL-C – high-density lipoprotein cholesterol; TG – triglycerides; TC – total cholesterol; LDL-C – low-density lipoprotein cholesterol; Basic – unadjusted model; Full – model adjusted for age, age^2^, sex, region of recruitment, height, education and smoking; P-value – level of statistical significance from crude and adjusted analysis; P-value* – level of statistical significance for a difference in each lipid fraction change between men and women recruited in the same year; M vs W – men versus women.
(DOCX)

Table S6 **Changes in lipids between 2004 and 2006 in the cross-sectional and prospective LIPIDOGRAM Studies – sensitivity analysis after exclusion of subjects on lipid lowering medication.** Changes between 2004 and 2006 are expressed as β-coefficients with respective standard errors (SE) from regression or generalized estimating equations-based models; HDL-C – high-density lipoprotein cholesterol; TG – triglycerides; TC – total cholesterol; LDL-C – low-density lipoprotein cholesterol; Basic – unadjusted model; Full – model adjusted for age, age^2^, sex, region of recruitment, height, education and smoking; P-value – level of statistical significance from basic or fully adjusted analysis.
(DOCX)

Table S7 **Association between mean changes in lipids and body mass index/waist circumference between 2004 and 2006 in LIPIODGRAM PLUS Study.** Data are expressed as means and standard errors of mean changes in each lipid fraction across quartile distribution of change in body mass index/waist circumference between 2004 and 2006, ranges of changes in obesity measures are shown per each quartile; TC – total cholesterol; HDL-C – high-density lipoprotein cholesterol; TG – triglycerides; P-value* – adjusted for age, sex, region of recruitment, height, education and smoking.
(DOCX)
