# Supplementary figures and images for: The Relation of Rapid Changes in Obesity Measures to Lipid Profile - Insights from a Nationwide Metabolic Health Survey in 444 Polish Cities
Source: PLoS One. 2014 Jan 31;9(1):e86837. doi: 10.1371/journal.pone.0086837 (PMC3908946; doi:10.1371/journal.pone.0086837)

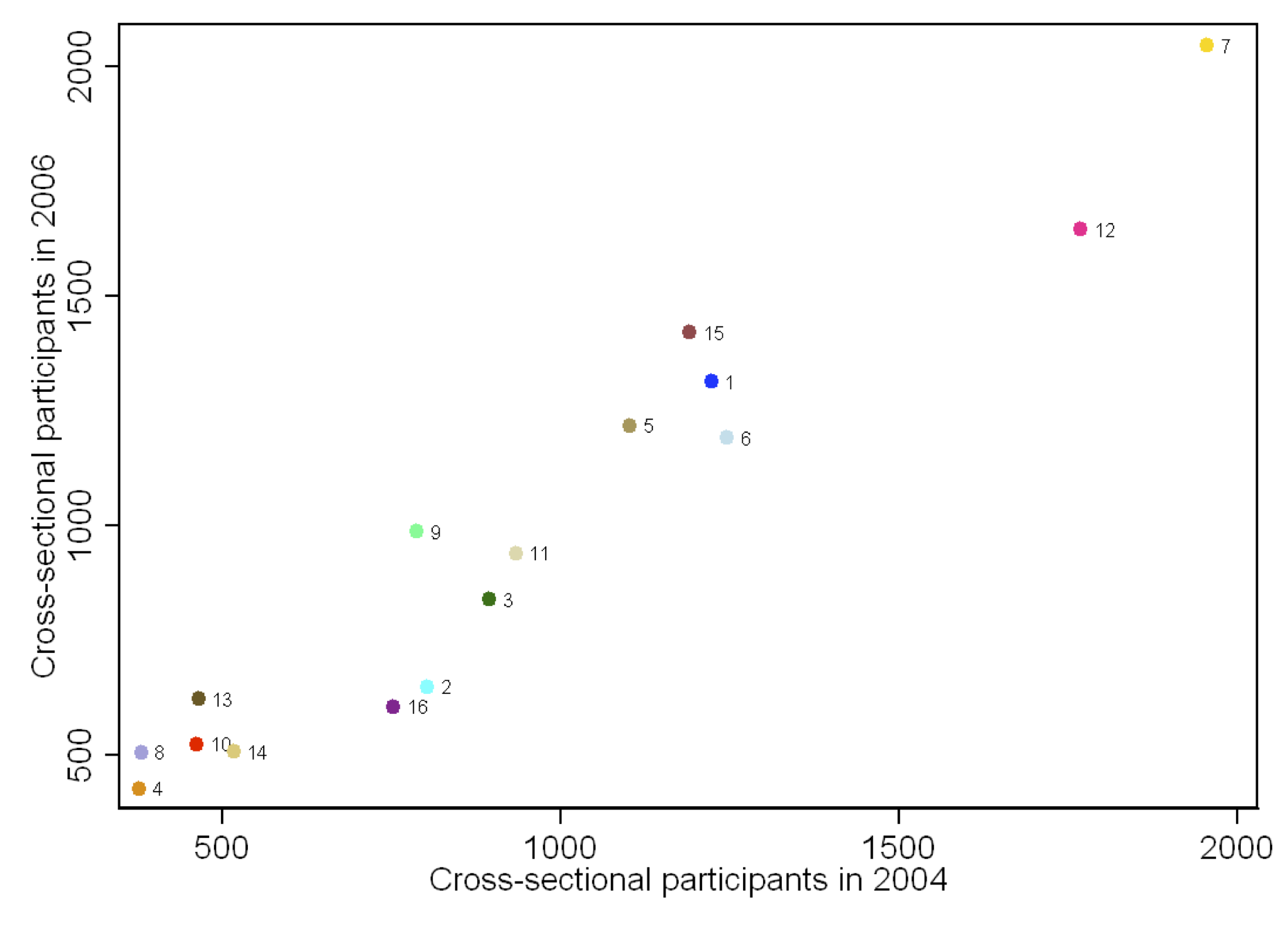

Supplement: Figure S1 — Correlation between numbers of individuals recruited in 2004 and 2006 into the cross-sectional LIPIDOGRAM Studies. (TIFF) [file pone.0086837.s001.tiff]

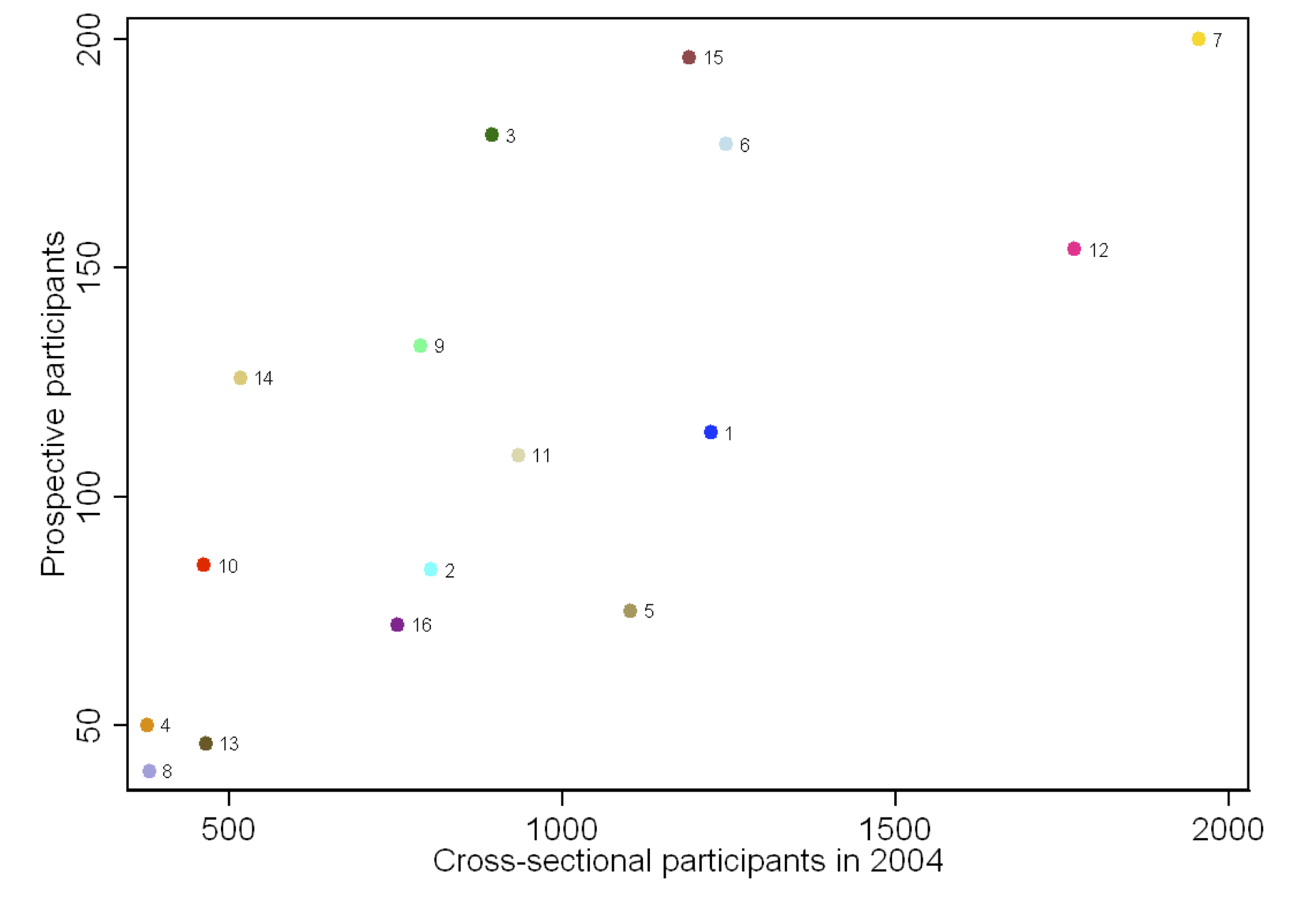

Supplement: Figure S2 — Correlation between numbers of individuals recruited in 2004 into the cross-sectional and prospective LIPIDOGRAM Studies. (TIFF) [file pone.0086837.s002.tiff]

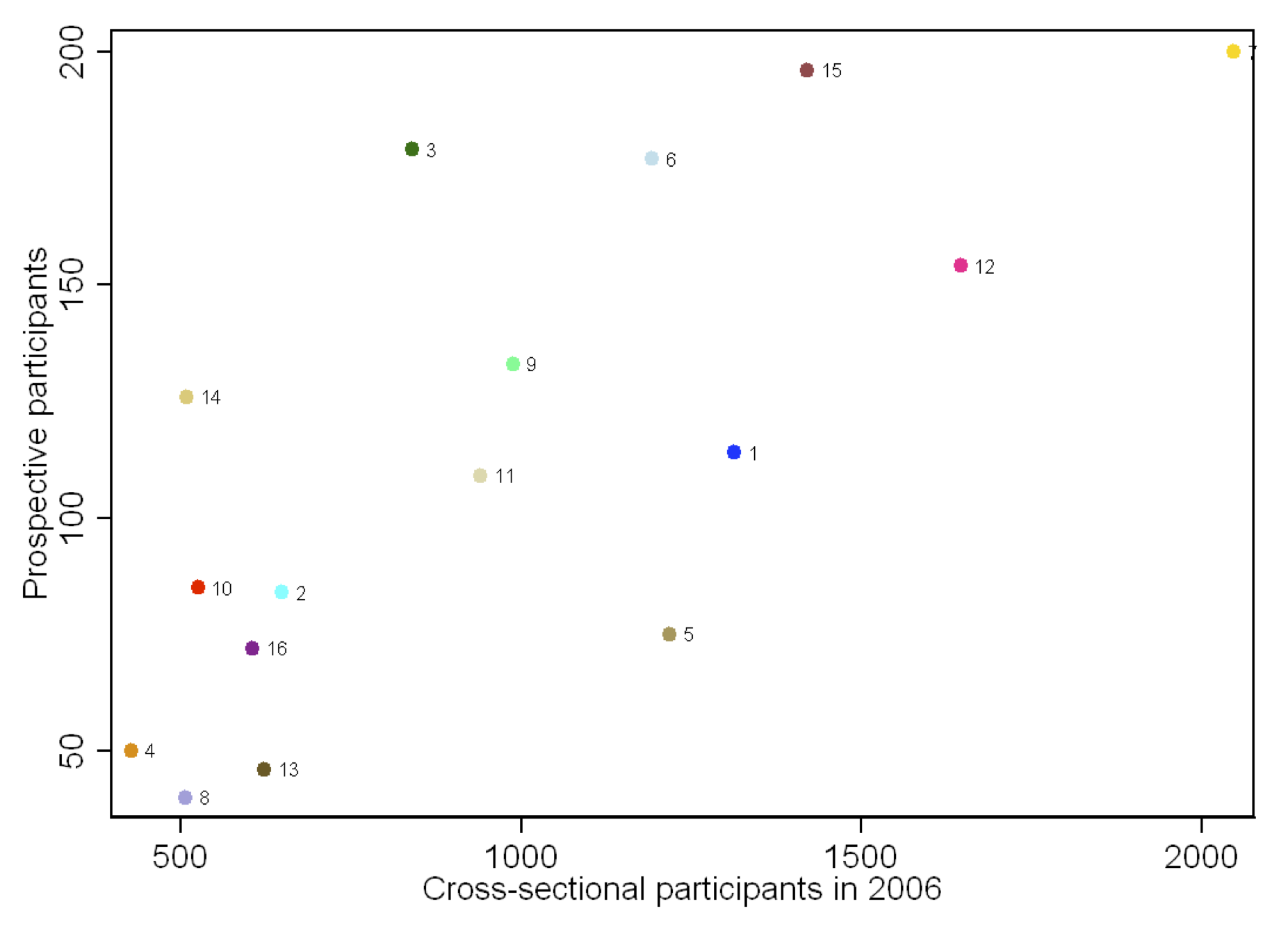

Supplement: Figure S3 — Correlation between numbers of individuals recruited in 2006 into cross-sectional and prospective LIPIDOGRAM Studies. (TIFF) [file pone.0086837.s003.tiff]

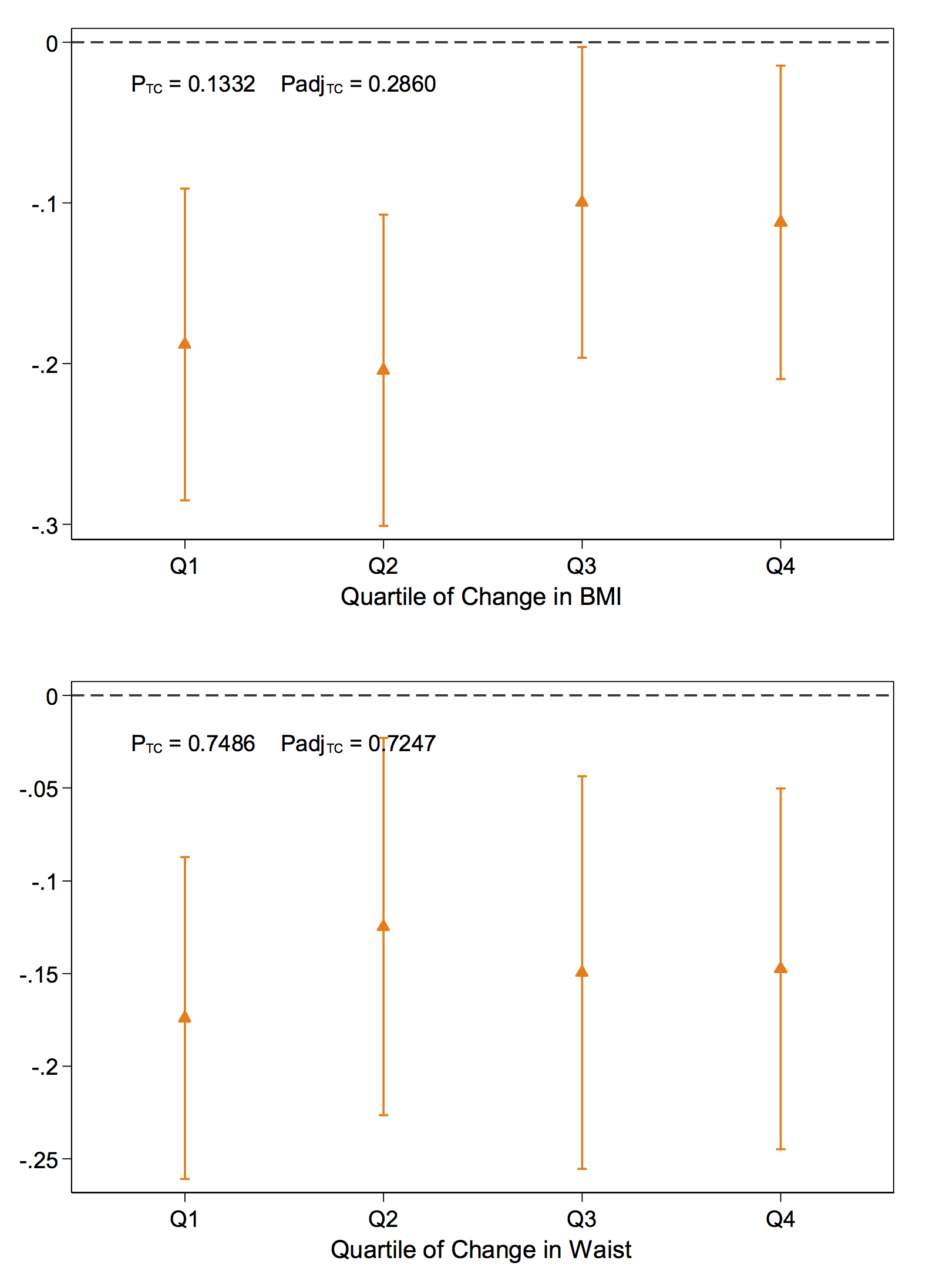

Supplement: Figure S4 — Mean changes in total cholesterol across 4 quartiles of changes in BMI (top panel) and waist circumference (bottom panel) between 2004 and 2006 in LIPIDOGRAM PLUS Study. Data are means and standard errors, the lowest quartile (Q1) - bottom 25th percentile of distribution in BMI or waist circumference increase between 2004 and 2006; P-values – level of statistical significance from test for linear trend; Padj - level of statistical significance (test for linear trend) after adjustment for age, sex, region of recruitment, height, education and smoking, the dotted horizontal line – no change. (TIFF) [file pone.0086837.s004.tiff]
