# Supplementary material for: The Relation of Rapid Changes in Obesity Measures to Lipid Profile - Insights from a Nationwide Metabolic Health Survey in 444 Polish Cities
Source: PLoS One. 2014 Jan 31;9(1):e86837. doi: 10.1371/journal.pone.0086837 (PMC3908946; doi:10.1371/journal.pone.0086837)
Supplement: Table S1 — Number of recruiting cities, physicians, recruited and included individuals in cross-sectional LIPIDOGRAM2004 and LIPIDOGRAM2006. Population data obtained from the Polish National Main Statistical Office (Glowny Urząd Statystyczny; GUS) –2003. (DOCX) [file pone.0086837.s005.docx]

| **Region** | **Population** | **(%)** | **LIPIDOGRAM2004** | | | | **LIPIDOGRAM2006** | | | |
| --- | --- | --- | --- | --- | --- | --- | --- | --- | --- | --- |
|  |  |  | **Cities** | **Doctors** | **Recruited** | **Included** | **Cities** | **Doctors** | **Recruited** | **Included** |
| **Dolnośląskie** | 2,898,313 | 7.6% | 34 | 52 | 1257 | 1222 | 31 | 42 | 1315 | 1314 |
| **Kujawsko-Pomorskie** | 2,068,142 | 5.4% | 24 | 36 | 834 | 802 | 21 | 30 | 650 | 649 |
| **Lubelskie** | 2,191,172 | 5.7% | 25 | 38 | 925 | 894 | 23 | 32 | 841 | 840 |
| **Lubuskie** | 1,008,786 | 2.6% | 12 | 17 | 398 | 377 | 11 | 14 | 428 | 428 |
| **Łódzkie** | 2,597,094 | 6.8% | 30 | 46 | 1167 | 1102 | 28 | 38 | 1219 | 1218 |
| **Małopolskie** | 3,252,949 | 8.5% | 38 | 58 | 1296 | 1246 | 34 | 47 | 1193 | 1192 |
| **Mazowieckie** | 5,135,109 | 13.5% | 60 | 92 | 1983 | 1956 | 55 | 75 | 2048 | 2047 |
| **Opolskie** | 1,055,667 | 2.8% | 12 | 18 | 395 | 379 | 10 | 16 | 509 | 507 |
| **Podkarpackie** | 2,097,248 | 5.5% | 24 | 36 | 808 | 787 | 22 | 31 | 988 | 988 |
| **Podlaskie** | 1,205,337 | 3.2% | 14 | 21 | 486 | 461 | 12 | 18 | 525 | 525 |
| **Pomorskie** | 2,188,918 | 5.7% | 25 | 39 | 965 | 933 | 23 | 32 | 942 | 941 |
| **Śląskie** | 4,714,982 | 12.4% | 56 | 85 | 1827 | 1768 | 50 | 68 | 1646 | 1646 |
| **Świętokrzyskie** | 1,292,334 | 3.4% | 15 | 23 | 491 | 464 | 13 | 19 | 624 | 623 |
| **Warmińsko- Mazurskie** | 1,428,552 | 3.7% | 16 | 25 | 549 | 516 | 14 | 21 | 508 | 508 |
| **Wielkopolskie** | 3,359,932 | 8.8% | 39 | 60 | 1241 | 1190 | 36 | 49 | 1422 | 1421 |
| **Zachodnio-pomorskie** | 1,696,073 | 4.4% | 20 | 29 | 782 | 752 | 19 | 24 | 607 | 606 |
| **In total** | 38,190,608 | 100% | 444 | 675 | 15404 | 14849 | 402 | 556 | 15465 | 15453 |
