# Supplementary material for: The Relation of Rapid Changes in Obesity Measures to Lipid Profile - Insights from a Nationwide Metabolic Health Survey in 444 Polish Cities
Source: PLoS One. 2014 Jan 31;9(1):e86837. doi: 10.1371/journal.pone.0086837 (PMC3908946; doi:10.1371/journal.pone.0086837)
Supplement: Table S2 — Individuals recruited into the prospective LIPIDOGRAM PLUS Study. (DOCX) [file pone.0086837.s006.docx]

| **Region** | **Recruited in 2004** | **Available for follow up in 2006** |
| --- | --- | --- |
| Dolnośląskie | 127 | 115 |
| Kujawsko-Pomorskie | 96 | 84 |
| Lubelskie | 195 | 179 |
| Lubuskie | 67 | 50 |
| Łódzkie | 93 | 75 |
| Małopolskie | 196 | 177 |
| Mazowieckie | 224 | 200 |
| Opolskie | 53 | 40 |
| Podkarpackie | 156 | 133 |
| Podlaskie | 99 | 85 |
| Pomorskie | 131 | 109 |
| Śląskie | 168 | 154 |
| Świętokrzyskie | 61 | 46 |
| Warmińsko- Mazurskie | 144 | 126 |
| Wielkopolskie | 216 | 196 |
| Zachodnio-pomorskie | 92 | 72 |
| In total | 2118 | 1841 |
