# Supplementary material for: The Relation of Rapid Changes in Obesity Measures to Lipid Profile - Insights from a Nationwide Metabolic Health Survey in 444 Polish Cities
Source: PLoS One. 2014 Jan 31;9(1):e86837. doi: 10.1371/journal.pone.0086837 (PMC3908946; doi:10.1371/journal.pone.0086837)
Supplement: Table S3 — Clinical characteristics of cross-sectional LIPIDOGRAM2004 and LIPIDOGRAM2006 Studies – sex-stratified analysis. Data are means and standard deviations, geometric means and standard deviations (triglycerides) or counts and percentages; BMI – body mass index; HDL-C – high-density lipoprotein cholesterol; TG – triglycerides; TC – total cholesterol; LDL-C – low-density lipoprotein cholesterol; treatment – lipid-lowering medication; P-value – level of statistical significance for comparison of men versus women. (DOCX) [file pone.0086837.s007.docx]

| **Characteristic** | **LIPIDOGRAM2004** | | | **LIPIDOGRAM2006** | | |
| --- | --- | --- | --- | --- | --- | --- |
|  | **Men** | **Women** | **P-value** | **Men** | **Women** | **P-value** |
| **n** | 6004 | 8845 | - | 5806 | 9647 | - |
| **Age (years)** | 54.7 (10.6) | 55.9 (10.7) | <0.001 | 54.9 (11.1) | 55.9 (11.1) | <0.001 |
| **Height (cm)** | 173.4 (6.9) | 161.7 (6.1) | <0.001 | 173.5 (6.5) | 161.7 (6.0) | <0.001 |
| **Weight (kg)** | 85.7 (13.9) | 73.2 (13.7) | <0.001 | 86.6 (13.9) | 73.6 (13.2) | <0.001 |
| **BMI (kg/m^2^)** | 28.5 (4.3) | 28.0 (5.1) | <0.001 | 28.8 (4.2) | 28.2 (4.9) | <0.001 |
| **Waist (cm)** | 97.1 (10.9) | 89.2 (13.4) | <0.001 | 98.5 (11.1) | 89.8 (12.9) | <0.001 |
| **HDL-C (mmol/L)** | 1.52 (0.36) | 1.74 (0.39) | <0.001 | 1.39 (0.34) | 1.61 (0.38) | <0.001 |
| **TG (mmol/L)** | 1.58 (0.74) | 1.42 (0.65) | <0.001 | 1.61 (0.69) | 1.48 (0.61) | <0.001 |
| **TC (mmol/L)** | 5.64 (1.11) | 5.85 (1.12) | <0.001 | 5.53 (1.11) | 5.74 (1.13) | <0.001 |
| **LDL-C (mmol/L)** | 3.32 (0.94) | 3.39 (0.95) | <0.001 | 3.33 (0.97) | 3.39 (1.0) | <0.001 |
| **Smokers (%)** | 1504 (25.1) | 1622 (18.3) | <0.001 | 1384 (23.8) | 1495 (15.5) | <0.001 |
| **Treatment (%)** | 1904 (31.7) | 2651 (30.0) | 0.02 | 1857 (32.0) | 3041 (31.5) | 0.55 |
