# Supplementary material for: The Relation of Rapid Changes in Obesity Measures to Lipid Profile - Insights from a Nationwide Metabolic Health Survey in 444 Polish Cities
Source: PLoS One. 2014 Jan 31;9(1):e86837. doi: 10.1371/journal.pone.0086837 (PMC3908946; doi:10.1371/journal.pone.0086837)
Supplement: Table S4 — Clinical characteristics of LIPIDOGRAM PLUS Study – sex-stratified analysis. Data are means and standard deviations, geometric means and standard deviations (triglycerides) or counts and percentages; BMI – body mass index; HDL-C – high-density lipoprotein cholesterol; TG – triglycerides; TC – total cholesterol; LDL-C – low-density lipoprotein cholesterol; treatment – lipid-lowering medication; P-value – level of statistical significance for comparison men versus women. (DOCX) [file pone.0086837.s008.docx]

| **Characteristic** | **2004** | | | **2006** | | |
| --- | --- | --- | --- | --- | --- | --- |
|  | **Men** | **Women** | **P-value** | **Men** | **Women** | **P-value** |
| **n** | 712 | 1128 |  | 712 | 1128 |  |
| **Age (years)** | 53.0 (9.5) | 53.5 (9.8) | 0.27 | 55.0 (9.5) | 55.5 (9.8) | - |
| **Height (cm)** | 174.0 (6.8) | 162.1 (5.8) | <0.001 | 174.0 (6.8) | 162.1 (5.8) | - |
| **Weight (kg)** | 86.9 (13.1) | 72.3 (13.1) | <0.001 | 88.0 (13.4) | 73.1 (13.1) | <0.001 |
| **BMI (kg/m^2^)** | 28.7 (3.9) | 27.5 (4.9) | <0.001 | 29.1 (4.0) | 27.8 (4.8) | <0.001 |
| **Waist (cm)** | 97.6 (9.9) | 87.2 (12.3) | <0.001 | 98.4 (10.1) | 88.2 (12.2) | <0.001 |
| **HDL-C (mmol/L)** | 1.51 (0.36) | 1.79 (0.39) | <0.001 | 1.37 (0.32) | 1.64 (0.37) | <0.001 |
| **TG (mmol/L)** | 1.64 (0.78) | 1.37 (0.63) | <0.001 | 1.71 (0.74) | 1.41 (0.58) | <0.001 |
| **TC (mmol/L)** | 5.73 (1.14) | 5.88 (1.16) | 0.005 | 5.56 (1.12) | 5.74 (1.13) | <0.001 |
| **LDL-C (mmol/L)** | 3.38 (0.97) | 3.40 (0.99) | 0.71 | 3.34 (0.97) | 3.40 (1.0) | 0.22 |
| **Smokers (%)** | 160 (22.5) | 201 (17.8) | 0.02 | 150 (21.1) | 167 (14.8) | <0.001 |
| **Treatment (%)** | 210 (29.5) | 302 (26.8) | 0.21 | 296 (41.6) | 410 (36.3) | 0.03 |
