# Supplementary material for: The Relation of Rapid Changes in Obesity Measures to Lipid Profile - Insights from a Nationwide Metabolic Health Survey in 444 Polish Cities
Source: PLoS One. 2014 Jan 31;9(1):e86837. doi: 10.1371/journal.pone.0086837 (PMC3908946; doi:10.1371/journal.pone.0086837)
Supplement: Table S5 — Changes in metabolic parameters between 2004 and 2006 in the LIPIDOGRAM studies – sex-stratified analysis. Changes between 2004 and 2006 are expressed as β-coefficients (β) with respective standard errors (SE) from linear regression or generalized estimation equations-based models; BMI – body mass index; HDL-C – high-density lipoprotein cholesterol; TG – triglycerides; TC – total cholesterol; LDL-C – low-density lipoprotein cholesterol; Basic – unadjusted model; Full – model adjusted for age, age2, sex, region of recruitment, height, education and smoking; P-value – level of statistical significance from crude and adjusted analysis; P-value* – level of statistical significance for a difference in each lipid fraction change between men and women recruited in the same year; M vs W – men versus women. (DOCX) [file pone.0086837.s009.docx]

| **Phenotype** | **Model** | **Cross-sectional** | | | | | **Prospective** | | | | |
| --- | --- | --- | --- | --- | --- | --- | --- | --- | --- | --- | --- |
|  |  | **Men** | | **Women** | | **M vs W** | **Men** | | **Women** | | **M vs W** |
|  |  | **β (SE)** | **P-value** | **β (SE)** | **P-value** | **adjusted P-value*** | **β (SE)** | **P-value** | **β (SE)** | **P-value** | **adjusted P-value*** |
| **BMI**  **(kg/m^2^)** | **Basic** | 0.27  (0.08) | <0.001 | 0.18  (0.07) | 0.02 | 0.41 | 0.37  (0.07) | <0.001 | 0.31  (0.06) | <0.001 | 0.45 |
|  | **Full** | 0.26  (0.08) | <0.001 | 0.15  (0.07) | 0.03 | 0.28 | 0.36  (0.08) | <0.001 | 0.12  (0.07) | 0.078 | 0.02 |
| **Waist**  **(cm)** | **Basic** | 1.37  (0.21) | <0.001 | 0.66  (0.20) | <0.001 | 0.012 | 0.79  (0.24) | <0.001 | 0.95  (0.20) | <0.001 | 0.61 |
|  | **Full** | 1.30  (0.20) | <0.001 | 0.62  (0.18) | <0.001 | 0.0012 | 0.41  (0.25) | 0.10 | 0.21 (0.22) | 0.34 | 0.53 |
| **HDL-C (mmol/L)** | **Basic** | -0.127 (0.006) | <0.001 | -0.125  (0.006) | <0.001 | 0.81 | -0.141  (0.009) | <0.001 | -0.151  (0.008) | <0.001 | 0.40 |
|  | **Full** | -0.126  (0.006) | <0.001 | -0.125  (0.006) | <0.001 | 0.91 | -0.141  (0.010) | <0.001 | -0.148  (0.008) | <0.001 | 0.58 |
| **TG**  **(mmol/L)** | **Basic** | 0.016 (0.008) | 0.05 | 0.038  (0.006) | 0.04 | 0.035 | 0.039  (0.015) | 0.010 | 0.030  (0.011) | 0.008 | 0.64 |
|  | **Full** | 0.018 (0.008) | 0.03 | 0.042  (0.006) | 0.02 | 0.021 | 0.048  (0.016) | 0.002 | 0.013  (0.012) | 0.28 | 0.07 |
| **TC**  **(mmol/L)** | **Basic** | -0.108 (0.02) | <0.001 | -0.111  (0.017) | <0.001 | 0.92 | -0.165  (0.037) | <0.001 | -0.142  (0.033) | <0.001 | 0.65 |
|  | **Full** | -0.1  (0.02) | <0.001 | -0.098  (0.016) | <0.001 | 0.92 | -0.139  (0.038) | <0.001 | -0.177  (0.033) | <0.001 | 0.46 |
| **LDL-C (mmol/L)** | **Basic** | 0.017  (0.018) | 0.33 | 0.000  (0.014) | 0.98 | 0.44 | -0.043  (0.032) | 0.18 | 0.001  (0.029) | 0.96 | 0.30 |
|  | **Full** | 0.023  (0.018) | 0.19 | 0.011  (0.014) | 0.46 | 0.57 | -0.026  (0.033) | 0.44 | -0.026  (0.029) | 0.37 | 0.96 |
