# Supplementary material for: The Relation of Rapid Changes in Obesity Measures to Lipid Profile - Insights from a Nationwide Metabolic Health Survey in 444 Polish Cities
Source: PLoS One. 2014 Jan 31;9(1):e86837. doi: 10.1371/journal.pone.0086837 (PMC3908946; doi:10.1371/journal.pone.0086837)
Supplement: Table S6 — Changes in lipids between 2004 and 2006 in the cross-sectional and prospective LIPIDOGRAM Studies – sensitivity analysis after exclusion of subjects on lipid lowering medication. Changes between 2004 and 2006 are expressed as β-coefficients with respective standard errors (SE) from regression or generalized estimating equations-based models; HDL-C – high-density lipoprotein cholesterol; TG – triglycerides; TC – total cholesterol; LDL-C – low-density lipoprotein cholesterol; Basic – unadjusted model; Full – model adjusted for age, age2, sex, region of recruitment, height, education and smoking; P-value – level of statistical significance from basic or fully adjusted analysis. (DOCX) [file pone.0086837.s010.docx]

| **Lipid fraction** | **Model** | **Cross-sectional** | | **Prospective** | |
| --- | --- | --- | --- | --- | --- |
|  |  | **β (SE)** | **P-value** | **β (SE)** | **P-value** |
| **HDL-C (mmol/L)** | **Basic** | -0.119 (0.005) | <0.001 | -0.141 (0.008) | <0.001 |
|  | **Full** | -0.122 (0.005) | <0.001 | -0.139 (0.008) | <0.001 |
| **TG (mmol/L)** | **Basic** | 0.034 (0.006) | <0.001 | 0.059 (0.012) | <0.001 |
|  | **Full** | 0.036 (0.006) | <0.001 | 0.050 (0.012) | <0.001 |
| **TC (mmol/L)** | **Basic** | -0.074 (0.015) | <0.001 | -0.039 (0.027) | 0.14 |
|  | **Full** | -0.066 (0.015) | <0.001 | -0.063 (0.027) | 0.021 |
| **LDL-C (mmol/L)** | **Basic** | 0.033 (0.013) | 0.01 | 0.065 (0.023) | 0.005 |
|  | **Full** | 0.042 (0.013) | 0.001 | 0.044 (0.024) | 0.06 |
