# Supplementary material for: The Relation of Rapid Changes in Obesity Measures to Lipid Profile - Insights from a Nationwide Metabolic Health Survey in 444 Polish Cities
Source: PLoS One. 2014 Jan 31;9(1):e86837. doi: 10.1371/journal.pone.0086837 (PMC3908946; doi:10.1371/journal.pone.0086837)
Supplement: Table S7 — Association between mean changes in lipids and body mass index/waist circumference between 2004 and 2006 in LIPIODGRAM PLUS Study. Data are expressed as means and standard errors of mean changes in each lipid fraction across quartile distribution of change in body mass index/waist circumference between 2004 and 2006, ranges of changes in obesity measures are shown per each quartile; TC – total cholesterol; HDL-C – high-density lipoprotein cholesterol; TG – triglycerides; P-value* – adjusted for age, sex, region of recruitment, height, education and smoking. (DOCX) [file pone.0086837.s011.docx]

| **BMI (kg/m^2^)** | **1 quartile**  **(-11.1 – -0.62)** | **2 quartile**  **(-0.62 – 0.34)** | **3 quartile**  **(0.35 – 1.26)** | **4 quartile**  **(1.26 – 11.27)** | **P-value** | **adjusted**  **P-value*** |
| --- | --- | --- | --- | --- | --- | --- |
| **TC (mmol/L)** | -0.188 (0.049) | -0.204 (0.049) | -0.100 (0.049) | -0.112 (0.050) | 0.13 | 0.29 |
| **HDL-C (mmol/L)** | -0.108 (0.012) | -0.150 (0.012) | -0.170 (0.012) | -0.160 (0.012) | 0.001 | 0.001 |
| **TG (mmol/L)** | -0.062 (0.033) | -0.017 (0.033) | 0.059 (0.033) | 0.124 (0.033) | <0.001 | <0.001 |
| **Waist (cm)** | **1 quartile**  **(-28 – -2)** | **2 quartile**  **(-1 – 0)** | **3 quartile**  **(1 – 4)** | **4 quartile**  **(5 – 29)** | **P-value** | **adjusted**  **P-value*** |
| **TC (mmol/L)** | -0.174 (0.044) | -0.125 (0.052) | -0.149 (0.054) | -0.147 (0.050) | 0.75 | 0.72 |
| **HDL-C (mmol/L)** | -0.134 (0.011) | -0.145 (0.013) | -0.152 (0.013) | -0.161 (0.012) | 0.09 | 0.09 |
| **TG (mmol/L)** | 0.030 (0.029) | -0.028 (0.035) | 0.096 (0.036) | 0.085 (0.033) | 0.001 | 0.003 |
